# Charged Domain Wall and Polar Vortex Topologies in a Room-Temperature Magnetoelectric Multiferroic Thin Film

**DOI:** 10.1021/acsami.1c17383

**Published:** 2022-01-19

**Authors:** Kalani Moore, Eoghan N. O’Connell, Sinéad M. Griffin, Clive Downing, Louise Colfer, Michael Schmidt, Valeria Nicolosi, Ursel Bangert, Lynette Keeney, Michele Conroy

**Affiliations:** † Department of Physics, Bernal Institute, School of Natural Sciences, University of Limerick, Limerick V94 T9PX, Ireland; ‡ Materials Sciences Division, Lawrence Berkeley National Laboratory, Berkeley, California 94720, United States; § Molecular Foundry, Lawrence Berkeley National Laboratory, Berkeley, California 94720, United States; ∥ Advanced Microscopy Laboratory & AMBER, Trinity College Dublin, Dublin D02 PN40, Ireland; ⊥ Tyndall National Institute, University College Cork, Cork T12 R5CP, Ireland; # School of Chemistry, Trinity College Dublin, Dublin D02 PN40, Ireland; ∇ Department of Materials, Imperial College London, Exhibition Road, London SW7 2AZ, U.K.; ○ London Centre for Nanotechnology, Imperial College London, Exhibition Road, London SW7 2AZ, U.K.

**Keywords:** multiferroic, polar, domain walls, topologies, vortex, thin film

## Abstract

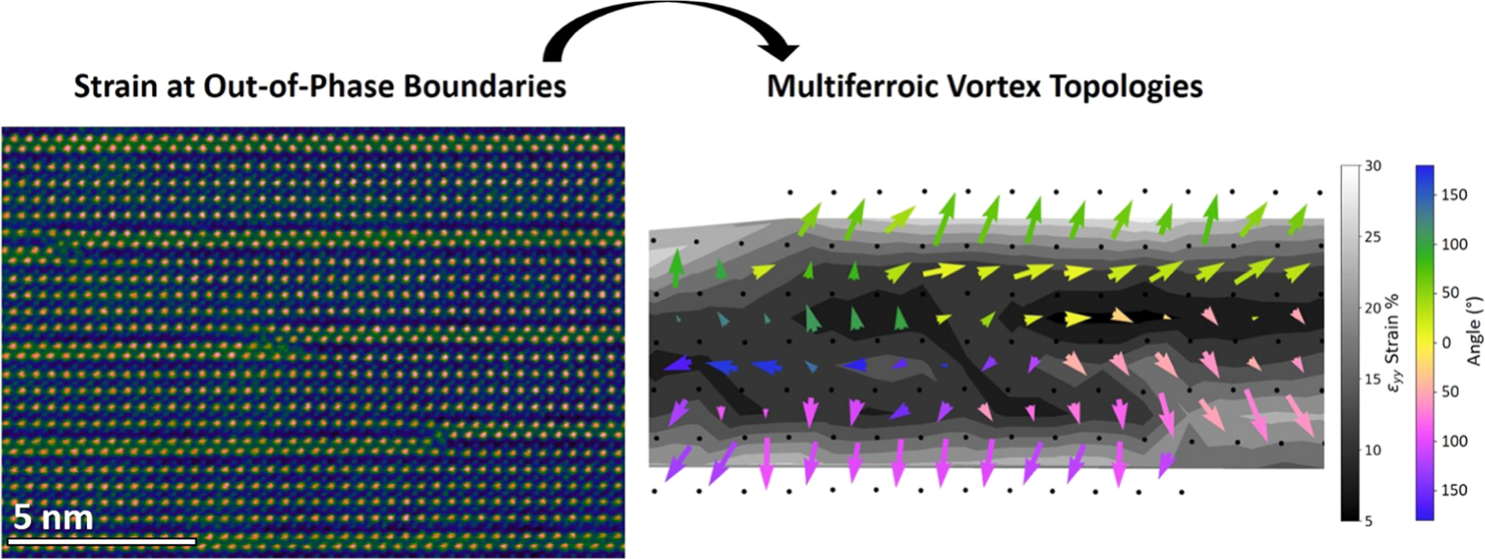

Multiferroic topologies are an emerging solution for future low-power magnetic nanoelectronics due to their combined tuneable functionality and mobility. Here, we show that in addition to being magnetoelectric multiferroic at room temperature, thin-film Aurivillius phase Bi_6_Ti*_x_*Fe*_y_*Mn*_z_*O_18_ is an ideal material platform for both domain wall and vortex topology-based nanoelectronic devices. Utilizing atomic-resolution electron microscopy, we reveal the presence and structure of 180°-type charged head-to-head and tail-to-tail domain walls passing throughout the thin film. Theoretical calculations confirm the subunit cell cation site preference and charged domain wall energetics for Bi_6_Ti*_x_*Fe*_y_*Mn*_z_*O_18_. Finally, we show that polar vortex-type topologies also form at out-of-phase boundaries of stacking faults when internal strain and electrostatic energy gradients are altered. This study could pave the way for controlled polar vortex topology formation via strain engineering in other multiferroic thin films. Moreover, these results confirm that the subunit cell topological features play an important role in controlling the charge and spin state of Aurivillius phase films and other multiferroic heterostructures.

## Introduction

Room-temperature multiferroic materials, possessing coupled ferroelectric and ferromagnetic states, have exciting potential for use in future low-energy data-storage devices such as magnetoelectric spin orbit logics for recurring neural networks.^1,2^ No such commercial devices presently exist, however, as single-phase multiferroic materials are extremely rare, due to the fundamental contraindication between ferroelectricity (empty d^0^ electronic structures) and ferromagnetism (occupied d*^n^* electronic structures).^3^ Layered oxide thin films, such as Bi_2_O_2_(A_*m*–1_B*_m_*O_3*m*+1_) Aurivillius phases, offer a flexible template to circumvent this, by accommodating differing types of A-site and B-site cations, to drive both ferroelectricity and ferromagnetism within the same structural phase.^4^ Here, *m* is the number of perovskite units interleaved between the (Bi_2_O_2_)^2+^ fluorite-type layers.^5,6^ Aurivillius phases are established ferroelectric materials with strong in-plane polarizations,^7^ high Curie temperatures (>600 °C), and fatigue-free energy storage performance.^8−10^ The rare demonstration of room-temperature ferromagnetism within a ferroelectric framework is achieved in Aurivillius phases with the introduction of magnetic ions within the scaffold.^11−15^ Our previous studies have shown that when B-site Ti (*x*) is maintained between 2.80 and 3.04, Fe (*y*) between 1.32 and 1.52, and Mn (*z*) between 0.54 and 0.64, thin-film samples on sapphire display saturation magnetization (*M*_S_) values as high as 215 emu/cm^3^, in-plane saturation polarization (*P*_s_) values of >26 μC/cm^2^, and demonstrate magnetoelectric switching at room temperature,^15−17^ as shown in Supporting Information Figure S1. Thin films prepared by both chemical solution deposition and direct liquid injection chemical vapor deposition (DLI-CVD)^18^ techniques demonstrate the room-temperature multiferroic behavior.

Direct piezoresponse force microscopy visualization of ferroelectric switching under the influence of a full in-plane magnetic field cycle demonstrated both irreversible and reversible magnetoelectric domain switching in Bi_6_Ti*_x_*Fe*_y_*Mn*_z_*O_18_ (B6TFMO).^16^ Great care was taken to perform detailed micro- and nanostructural analysis combined with rigorous statistical analysis, which concluded that ferromagnetic secondary phase impurities do not affect the measurements observed with a confidence level of ≥99.5%.^19^ This is an important conclusion, as without such rigorous analysis of sample purity, one cannot be confident that a material is truly a single-phase multiferroic.

B6TFMO can be thought of as a two-dimensional (2D) nanostructured framework, with five perovskite cells sandwiched between dielectric (Bi_2_O_2_)^2+^ layers. To maximize electrostatic interactions with the layer of oxygen anions in the (Bi_2_O_2_)^2+^ layer, the more highly charged Ti^4+^ cations partition close to the (Bi_2_O_2_)^2+^ layers.^20^ Lattice parameter differences between the unconstrained (Bi_2_O_2_)^2+^ layer (3.80 Å) and that of the unconstrained perovskite block (3.89 Å) put the perovskite blocks closest to the (Bi_2_O_2_)^2+^ layers under compressive stress.^21−23^ This combination of intrinsic elastic strain and elastic energy gradients drives the larger Mn cations toward the center-most layer of the structure in the *m* = 5 B6TFMO structure to diminish interlayer stress.^17^ Significantly, this magnetic cation partitioning increases the probability of nearest-neighbor magnetic interactions in the central layer by up to 90% compared to a case where the magnetic cations are randomly distributed over the five available B-sites. According to the Goodenough–Kanamari rules, Fe^3+^–O–Mn^4+^ or Mn^3+^–O–Mn^4+^ interactions and interactions of Mn^3+^–O–Mn^3+^ having longer bonds at the central layers are expected to show ferromagnetic coupling via double-exchange mechanisms.^24−26^ This magnetic cation partitioning is thus pivotal in explaining pathways to long-range magnetic order and the distinct room-temperature multiferroic properties of this tantalizing B6TFMO material system.^17^ This would indicate a superexchange-mediated mechanism for ferromagnetism in B6TFMO, as opposed to the canting of the collinear antiferromagnetic moments due to Dzyaloshinskii–Moria interactions to yield weak ferromagnetism in BiFeO_3_.^27,28^

As the multiferroic-dielectric naturally layered structure is at the subunit cell scale,^29,30^ probing any changes within this layered structure requires a characterization technique with atomic-scale spatial resolution. Aberration-corrected scanning transmission electron microscopy (STEM) allows the multiferroic research community to investigate domain wall (DW) topologies at the subatomic scale.^31^ Using STEM, we can quantify the atomic displacements and thus polarization changes at and within topologies. Recently, Campanini et al.,^32^ in a STEM-based paper, reported in-plane polarization and lateral domains within *m* = 4 Bi_5_FeTi_3_O_15_ Aurivillius thin films. No charged ferroelectric DWs were present and instead neutral ferroelectric DWs are located within the dielectric (Bi_2_O_2_)^2+^ layers. In contrast, we confirm by STEM characterization that charged 180° head–head (H–H) and tail–tail (T–T)-type DWs run through the entire film thickness of Mn containing B6TFMO thin film. This is the first study to experimentally report the atomic-scale structure and composition of charged-type DWs in a room-temperature multiferroic Aurivillius phase material. Perfectly parallel H–H/T–T 180° walls of B6TFMO were calculated to be energetically unfavorable by density functional theory (DFT). These first-principles calculations explain the observed angled orientation of the 180° DWs during imaging across all of the samples. DWs must bend to lower the energy cost while remaining in a charged state.

Additionally, we have found that polar vortex topologies were present in regions where out-of-phase boundary (OPB) defects are spaced between 5 and 8 perovskite cells apart. In the regions where OPBs and associated stacking default defects were present, the magnetic Mn and Fe ion partitioning increased. Theoretical reports predict that while OPBs can suppress ferroelectricity,^33^ their presence results in elevated magnetic ion interactions, thereby increasing the extent of long-range magnetic order.^34,35^ Thus, inducing higher OPB densities could result in improved magnetoelectric coupling and device efficiency. Within this study, we investigate the role these OPB defects play in the formation of charged DWs and polar vortices. We observe increased partitioning of lower valence cations toward the vortex core, indicating that there is an increased electrical conductivity at the ferroelectric vortex core. We map the picometer-scale atomic column shifts and unit cell deformation as demonstrated previously for other ferroic materials,^31^ revealing the direct link between OPBs and polar vortices in Aurivillius phase thin films. In addition to data-storage capabilities, this work demonstrates the wider technological potential of B6TFMO, with prospects for application in energy-efficient nanoelectronics and DW devices.

## Results and Discussion

**Figure 1:**
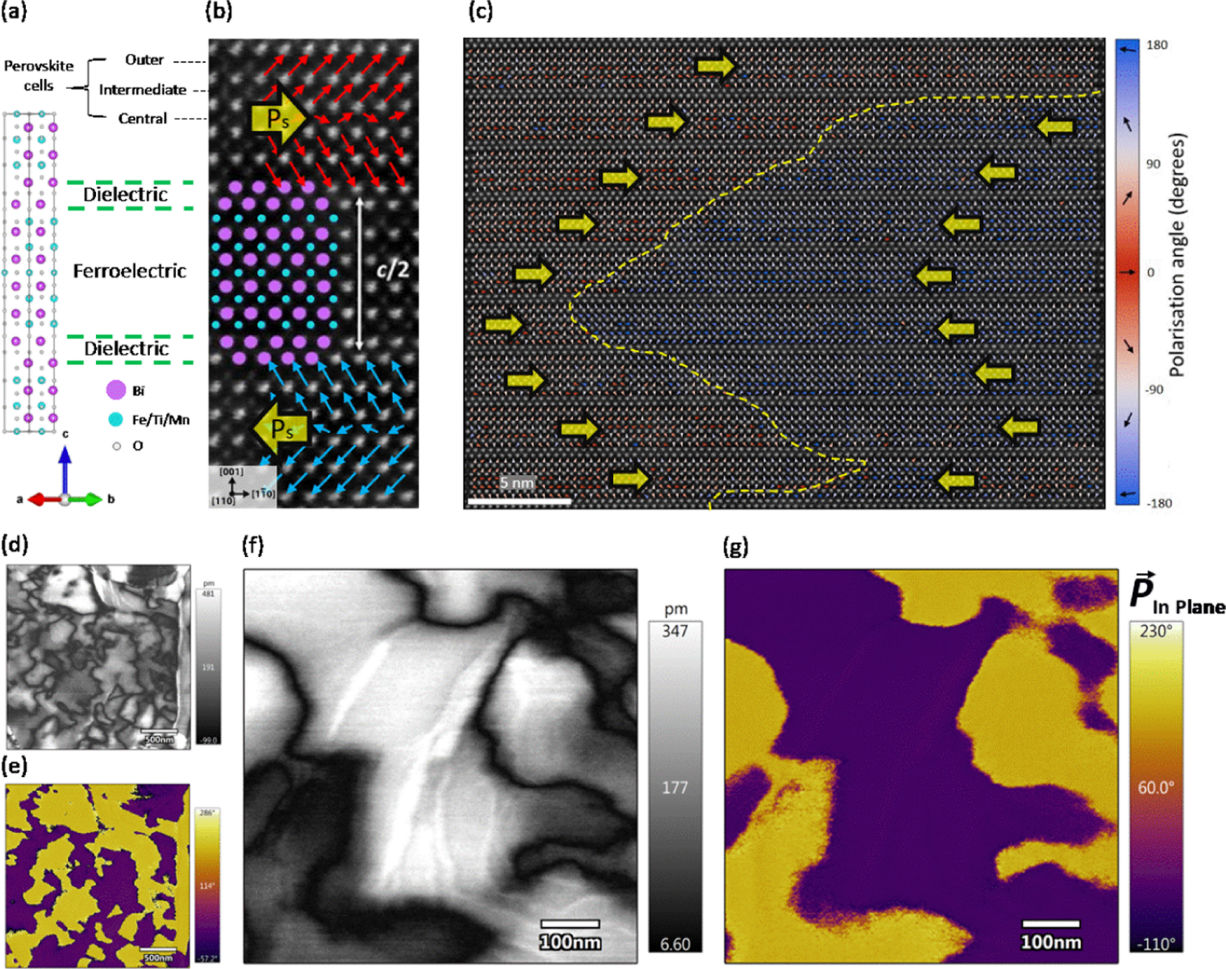
(a) Structural model of the five-layered Aurivillius phase B6TFMO unit cell (polar orthorhombic space group). One ferroelectric block with one dielectric layer corresponds to half of one Aurivillius phase unit cell. (b) A-site and B-site atoms in the B6TFMO structure are overlaid on an atomic-resolution high-angle annular dark-field image. Red/blue arrows are an illustration of the expected polarization of perovskite cells in the ferroelectric layer. The resulting in-plane net polarization (*P*_s_) is indicated by large yellow arrows. The ferroelectric (perovskite) and dielectric (fluorite) layers can clearly be distinguished by the Bi atom structures. (c) High-angle annular dark-field image overlaid with reverse B-site displacement polarization map of each perovskite cell. Polarization unit vectors are color-coded red/blue to illustrate the in-plane polarization direction. The approximate position of a head-to-head 180° DW is marked by the dashed yellow line. Scalebar = 5 nm. See Supporting Information Figures S2 and S3 for maps of the polarization averaged over 5 × 5 perovskite cells in the ferroelectric layer. In-plane piezoresponse force microscopy (PFM) (d) amplitude and (e) phase measurements of the sample surface demonstrate the magnitude of piezoresponse and polar orientation, respectively. A closer examination of PFM amplitude in panel (f) and PFM phase in panel (g) demonstrates that curved DWs separate oppositely orientated domains. Where these oppositely orientated polarizations have similar PFM amplitudes and meet 180° either at head-to-head or tail-to-tail configurations, we anticipate that the DW will be charged at that region.

The 100 nm thick Aurivillius films in this study were synthesized by liquid injection chemical vapor deposition and the magnetoelectric multiferroic properties were confirmed in our previous reports.^15,16^Figure 1 shows a model of the five-layered B6TFMO unit cell alongside an experimental confirmation of the atomic-resolution STEM high-angle annular dark-field (HAADF) image of the structure. One ferroelectric block with one dielectric layer corresponds to half of one (0.5) Aurivillius phase unit cell. The Aurivillius phase materials are established ferroelectrics, strongly favoring in-plane polarization, with spontaneous electrical polarization (*P*_s_) of ∼50 μC cm^–2^,^36^ as observed from previous macroscopic polarization *vs* electric-field measurements, piezoresponse force microscopy measurements, and *ab initio* calculations. In this contribution, we use HAADF-STEM cross-sectional imaging to investigate the polar behavior within sections of B6TFMO films. The large yellow arrows in Figure 1a and throughout this paper indicate the net polarization vector for each ferroelectric layer. The polarization vector was determined by the conventional approach of reverse B-site displacement mapping.^32,37^ The ferroelectric–dielectric layered structure within the B6TFMO unit cell leads to distinctive ferroelectric behavior, where oppositely polarized perovskite cells coexist side by side within the ferroelectric layer,^32^ as shown in Figure 1b. The perovskite cell is pseudo-cubic with a ferroelectric dipole forming analogous to that in BiFeO_3_. The outer perovskite cells bonded to the dielectric layer are hypertetragonal and thus highly strained along the *c*-axis (ε*_yy_*) compared to BiFeO_3_, BiTiO_3_, or PbTiO_3_ unit cells. At these outer perovskite cells, the polarization is larger and always points toward the dielectric layer. At the three perovskite cells in the center of the layer, the ε*_yy_* strain relaxes and thus the polarization state has a shallower potential well.^38^ In other words, with reduced ε*_yy_* strain present, the polarization direction is largely determined by electrostatic stress and has a lower energy cost for switching.

Figure 1c shows 180° nominally charged H–H (polarizations pointing directly toward each other) and T–T (polarizations pointing directly away from each other) domain walls in multiferroic B6TFMO, which pass throughout the 100 nm film thickness. Lateral PFM imaging (Figure 1d–g) reveals the curved nature of the domain walls at the surface and that oppositely orientated polarizations meet 180° either at H–H or T–T configurations along parts of the curved domain wall, similar to that observed through the film (Figure 1c). This observation for five-layered B6TFMO is different from observations so far for the *four-layered* Bi_5_Ti_3_FeO_15_ within a similar homologous series, where nominally charged domain walls were only stable when the film thickness was reduced to half a unit cell (∼2 nm thick), being gradually lost with increasing film thickness.^7^ The depth of the domain wall through the 100 nm thick film in odd-layered *m* = 5 B6TFMO indicates that ferroelectric blocks are vertically coupled, and unlike previous reports^32^ of the even-layered *m* = 4 Bi_5_Ti_3_FeO_15_ phase, the ferroelectric perovskite blocks are not fully electrostatically isolated by the dielectric (Bi_2_O_2_)^2+^ layers in between. This difference between *m = 4* and *5* phases is likely due to symmetry constraints. Polar Aurivillius phases having *even* number of *m* perovskite layers (e.g., *m* = 2 (SrBi_2_Ta_2_O_9_), *m* = 4 (Bi_5_Ti_3_O_15_), etc.) crystallize with orthorhombic (*A*2_1*am*_) symmetry and retain a mirror plane perpendicular to the *c*-axis. This prohibits out-of-plane polarization in even-layered Aurivillius phases and the polarization is confined to the lateral plane. Polar Aurivillius phases having *odd m* number of perovskite layers (e.g., *m* = 3 (Bi_4_Ti_3_O_12_), *m* = 5 (Bi_6_Ti_3_Fe_2_O_18_), etc.) crystallize to lower symmetry systems (e.g., *B*2*cb*, *B*2*eb*, *B*1a1), where retention of the mirror plane is not energetically favorable. This enables a minor out-of-plane polarization to exist in the *c*-direction for odd-layered phases, in conjunction with a major in-plane polarization. Indeed, vertical switching experiments, performed previously for these samples,^15,16^ demonstrate an out-of-plane ferroelectric response and vertical ferroelectric switching for *m* = 5 B6TFMO.

Lateral PFM imaging of our 100 nm thick B6TFMO films confirms the polar nature of the films at the surface. Figure 1d,e demonstrates that the films are naturally self-polarized and a random mixture of domain states is exhibited, separated by 180° DWs. As expected from the crystal symmetry of the *m* = 5 Aurivillius phases, having an odd *m* number of perovskite units, the polarization primarily lies along the lateral direction, with only minor polarization along the out-of-plane, vertical direction. A closer inspection of the domains and DW configurations in Figure 1f,g reveals the curved nature of DWs at the surface, similar to that observed through the film in Figure 1c. We also observe regions where oppositely orientated in-plane domains have similar magnitudes of piezoresponse. Hence, when these oppositely orientated polarizations meet 180° either at H–H or T–T (tail-to-tail) configurations along the parts of the curved DW, we anticipate that the DW will be charged at that region.

H–H DWs have “bound positive charge” from the adjacent polarizations, which should be screened by a negative surface charge density (σ_s_) equal to 2 × *P*_s_.^39^ In the case of B6TFMO where in-plane *P*_s_ = 0.5 C/m^2^,^36,40^
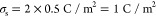
1To make sense of what this means on the atomic scale, we convert it to units of |e|/perovskite cell, where |e| = 1.6 × 10^–19^, or elementary charge, and perovskite cell is the average surface area of a perovskite cell, approximately 3.835 Å × 4.5 Å. Then,
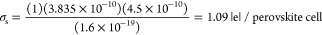
2This calculated σ_s_ is of the same order as that found at strongly charged DWs in other perovskites.^41^ As charge is likely to be spread across the DW width for 5–8 perovskite cells, rather than be confined to a 2D surface, a more realistic number for charge density (ρ) would be
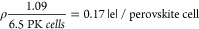
3However, this value would vary depending on the local DW width. The screening charge could be provided by either [Bi/Ti/Mn/Fe] vacancies or a local change in oxidation state. The existence of the two-dimensional sheet of charge carriers passing through layers of a multiferroic film raises the possibility of electroresistance complementing magnetoresistance in a multiferroic tunnel junction memory device.^42,43^

**Figure 2:**
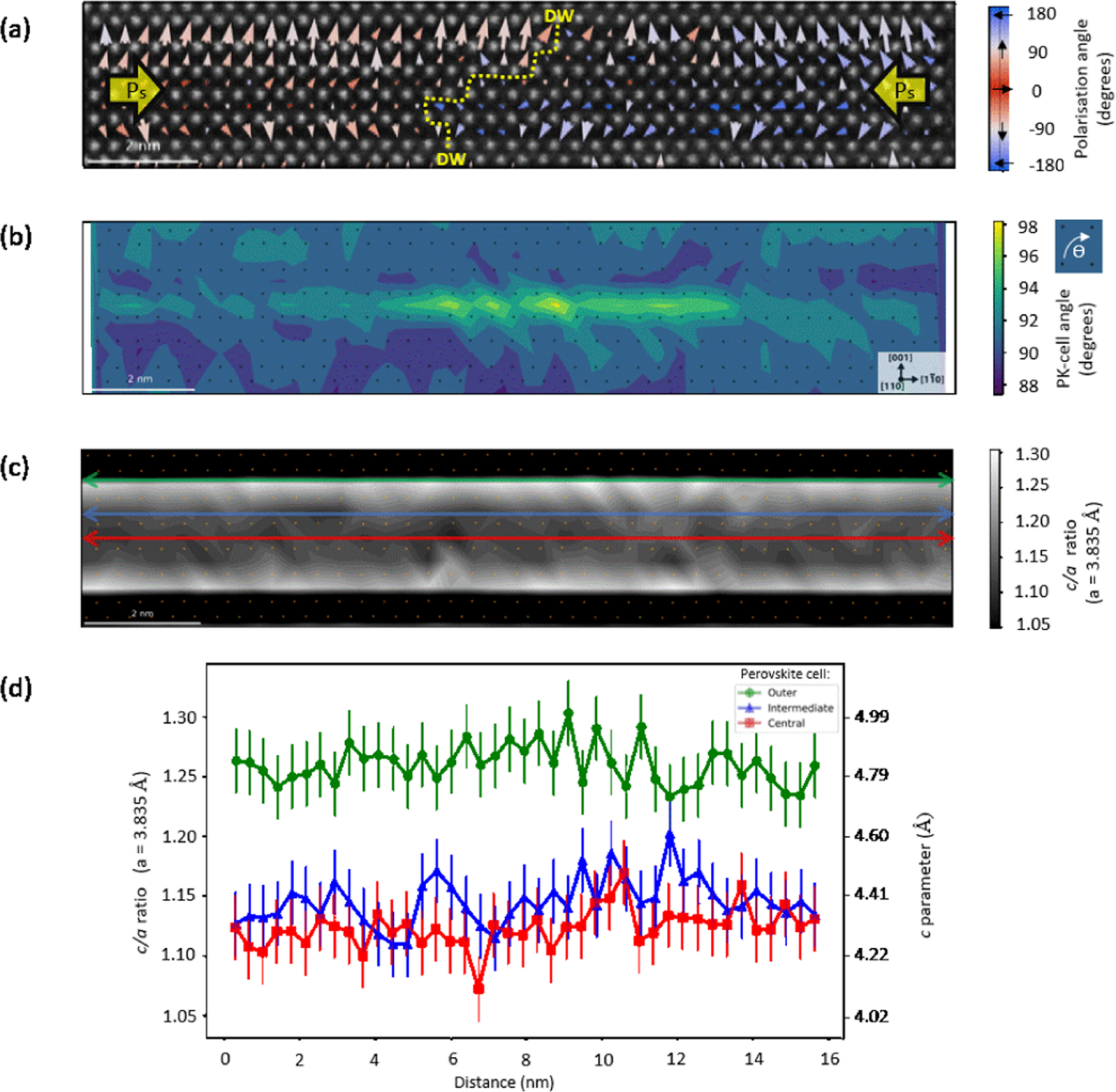
(a) Polarization map across a head-to-head charged DW. The arrow lengths indicate the relative magnitude of the B-site displacement and hence the relative magnitude of the polarization. A yellow dashed line indicates the approximate DW position. The polarization magnitudes are suppressed near the DW, indicating a width of approximately 5–8 perovskite cells. (b) Map of perovskite cell angles reveals a ∼6° shearing at the DW. (c) Map and (d) profiles of c/a ratio comparing the outer, intermediate, and central perovskite cells. The c/a ratio is measured with respect to the average *a* parameter of 3.835 Å, making c/a equal to strain ε*_yy_* + 1. Error bars represent the estimated measurement uncertainty. The measured *a* parameter varies ± 4% within the ferroelectric layer. This alters the actual measured c/a values from those on the graph, but introduces more error while at the same time not changing the relationship between outer, intermediate, and central perovskite cells. See Supporting Information Figure S3 for complete results. Scalebars = 2 nm.

Figure 2 displays the detail of the perovskite cell polarization evolution across the 180° H–H DW. The yellow traced domain wall position is approximated as running between the inflection point of the polarization in the outer perovskite cells. The 5–8 perovskite cells of inhomogeneous net polarization is similar in width to charged DWs in BiFeO_3_ but quite large for a ferroelectric domain wall.^44^ This infers a lower exchange energy allowed by a lower anisotropy cost. That is to say, the central perovskite cells seem to be more “polarizable” than structurally harder ferroelectrics with thinner DWs such as PbTiO_3_.^45^ The anisotropy cost of the DW is studied by examining rotation and ε*_yy_* strain maps in Figure 2b,c, respectively. While no clear change in ε*_yy_* strain was identified at the DW in Figure 2c, the strong rotation of ∼6° in Figure 2b indicates a shearing response of the structure at the DW. The same effect is seen in BiFeO_3_ H–T DWs.^44^ The lack of strain difference between domains confirms that there is little anisotropy cost, allowing for a semicontinuous rotation of the polarization i.e., Ising–Bloch like DW.^46^ The property of central layers being “polarizable”, specifically in ferroelectric–dielectric multilayers, demonstrates “structural softness” that is consistent with a large magnetoelectric effect.^47^ Furthermore, the relatively low anisotropy associated with the domain wall indicates that switching is determined only by the electrostatic energy, with no elastic energy cost. These results imply that charged DWs in B6TFMO are likely to be highly mobile with low dielectric loss.

If one applies the knowledge gained from BiFeO_3_ research, then, the lowest-energy polarization state is to point to one of the “corner” Bi atoms of the perovskite cell. This appears to contribute to the angled orientation of the DW, seen throughout the figures in this study. We performed first-principles calculations to understand the cation site preference and DW energetics for B6TFMO domains. Full calculation details are given in the methods; here, we summarize the main results.

**Figure 3:**
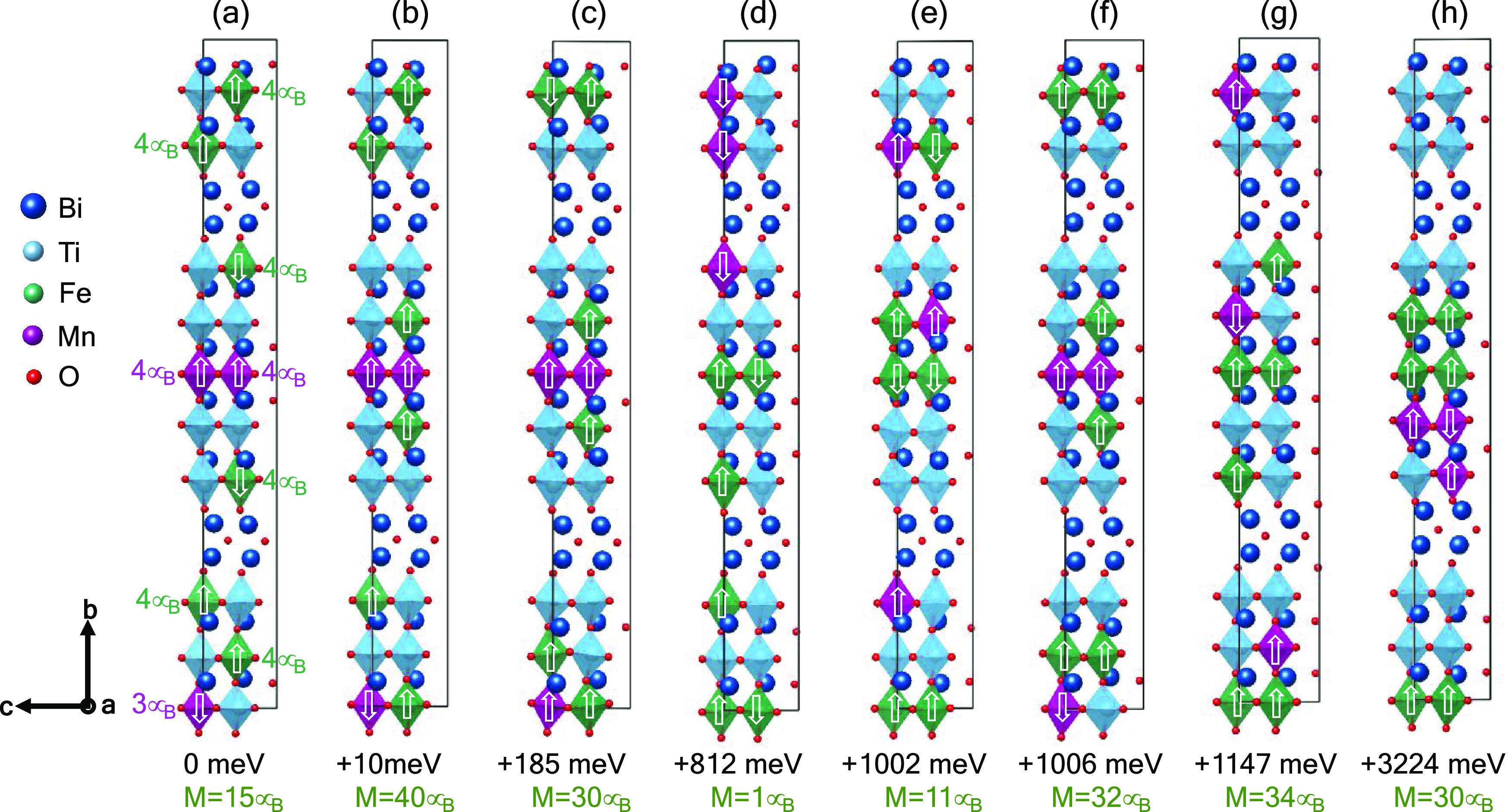
(a) Calculated Ti–Fe–Mn cation order structures using first-principles calculations for eight (a–h) different configurations. The crystal structures are fully optimized (lattice parameter and internal coordinates) for the cation ordering scheme considered. The calculated relative energy of various structures is given below each configuration relative to panel (a), which is set to 0 eV. The final calculated magnetic moment for the unit cell is also reported below each crystal structure. The final magnetic ordering for each structure is depicted with arrows on the Fe and Mn ions, with the local projected moments also shown for panel (a).

Our unit cell comprised Bi_24_Ti_11_Fe_6_Mn_3_O_72_, which most closely matched the stoichiometry of the experimental crystal B6TFMO-Bi_24_Ti_11.2_Fe_6.08_Mn_2.72_O_72_, while maintaining a feasible cell size for first-principles calculations. We fixed the “inner” perovskite units to be Bi–Fe–O (green shaded polyhedra) and Bi–Mn–O (magenta shaded polyhedra), as indicated in Figure 3, which is consistent with previous experimental and theoretical studies on cation ordering in Aurivillius phases.^35^ With this constraint, we explored the energetics of cation ordering for eight configurations of the remaining five cations, as illustrated in Figure 3. Our cation ordering energetics and resulting magnetic polarizations are also summarized in Figure 3. Overall, we find the lowest-energy cation orders to have Mn ions in the “inner” perovskite units—the lowest-energy configuration with Fe in the inner units (Figure 3d) is over 800 meV per unit cell higher in energy than the lowest-energy configuration with Mn in the inner units (Figure 3a). These results are consistent with previous STEM experiments,^17^ which demonstrated a clear preference for Mn cations to partition into the central perovskite layer but no significant preference for Fe to partition to the inner layers. Aside from the inner perovskite unit chemistry, we find that the preferred cation ordering is where the magnetic cations (Fe and Mn) are separated, i.e., they share minimal common oxygen bonds. This general trend can be observed throughout the eight cation configurations considered—the lowest-energy structures comprise of separated magnetic (Fe and Mn) cations, whereas the most unstable structures have clustered magnetic cations. For example, the highest-energy configuration, Figure 3h, comprises of Fe and Mn atoms that are clustered together with the outer blocks comprising mostly of Ti–O polyhedra. The clustering of Fe and Mn in the central layer results in more significant polyhedral tilting and stretching compared to when the magnetic cations are more spread out—this results in energy destabilizing tilting and polyhedral distortions that result in this higher-energy structure. Tilting and rotation of the octahedra are common features of the ferroelectric phase transitions in the Aurivillius phases and are energetically more favorable than octahedral deformations. All of our considered configurations have ferrimagnetic ordering with a net magnetic moment ranging from 1μ_B_ to 40μ_B_ per unit cell, depending on the particular cation ordering.

The mechanism involving ligand orbitals to facilitate coupling between metal electrons is referred to as superexchange. According to the Goodenough–Kanamori rule,^25^ superexchange interactions are antiferromagnetic where virtual electron transfer is between overlapping orbitals that are each half-filled, for example, between the e_g_ orbitals in high-spin Fe^3+^. Previous *ab initio* work on Bi_5_Ti_3_FeO_15_ Aurivillius phases (with iron as the only magnetic cation) found superexchange-driven antiferromagnetic ordering^35^ with a corresponding zero or low total magnetization. On the other hand, the Goodenough–Kanamori rule predicts that superexchange interactions are strong and ferromagnetic when virtual electron transfer is from a half-filled orbital to an empty orbital, for example, between a half-filled e_g_ orbital in high-spin Fe^3+^ and an empty e_g_ orbital in low-spin Mn^3+^ or in Mn^4+^ where e_g_ orbitals are always empty regardless of the extent of the octahedral crystal field splitting. Furthermore, ferromagnetic coupling of Mn^3+^–O–Mn^3+^ is also possible via semicovalent exchange, particularly for Mn^3+^–O–Mn^3+^ interactions at the central Aurivillius phase layers having longer bonds.^24^ In the mixed transition-metal phase B6TFMO, we find that the central Mn–Mn bonds favor ferromagnetic ordering. Therefore, we find that the inclusion of Mn in these materials promotes ferromagnetic ordering, with higher net magnetization than the Fe-only case. Importantly, as summarized in Figure 3, particular magnetic exchange interactions and resulting magnetization are highly dependent on the cation site order. Indeed, experimentally, we observe^17^ a marked preference for magnetic cations to partition to the central perovskite layers of the B6TFMO structure. This is key to explaining pathways to long-range magnetic order in the unique room-temperature multiferroic material system B6TFMO.

Finally, we calculate the domain wall energetics of two configurations—(i) H–H in-plane (i.e., opposite electronic polarizations in *c*-direction) and (ii) head-to-tail in the out-of-plane direction (i.e., opposite electronic polarizations in the *b-*direction). We consider domains of double stoichiometry in the *c*-direction and fix the central atoms in each domain for each DW relaxation. We find the H–H DW (i) to be very energetically unfavorable with a cost of 260 meV/formula unit compared to the head-to-tail DW (ii). Therefore, we find that abrupt 90° DWs are highly unfavorable in this class of oxides. This is consistent with the experimental observations, which have a staggered domain wall to avoid highly unfavorable H–H DWs, as expected from electrostatics.

The structural mismatch between the lattice parameter (*a*) of the (Bi_2_O_2_)^2+^ layer (*a* = 3.80 Å) and that of the perovskite block (*a*_p_ = 3.89 Å, where *p* denotes pseudo)^48^ imparts an elastic strain energy gradient on the layered Aurivillius system. This structural mismatch puts the perovskite blocks under compression, and the bismuth oxide layers under tension. Bulk compressibility calculations indicate that the total elastic strain energy, *E*, caused by the dilation of (Bi_2_O_2_)^2+^ unit and the compression of perovskite-like units increases with the number of *m* perovskite layers.^21^ The outer perovskite blocks closest to the (Bi_2_O_2_)^2+^ layers are under the most compressive stress, with perovskite blocks at the center of the structure experiencing the most relief from this stress. The measured *a* spacing in B6TFMO ranges from 3.68 to 3.99 Å through the layers. The average *a* value measured (3.835 Å) agrees exactly with previous Aurivillius phase measurements,^48^ falling between the natural dielectric layer and perovskite cell *a* values.^22^ Therefore, the outer perovskite cells bonded to the dielectric layer are effectively under compressive epitaxial stress/strain (−ε*_xx_*) for their *a* parameter.

**Figure 4:**
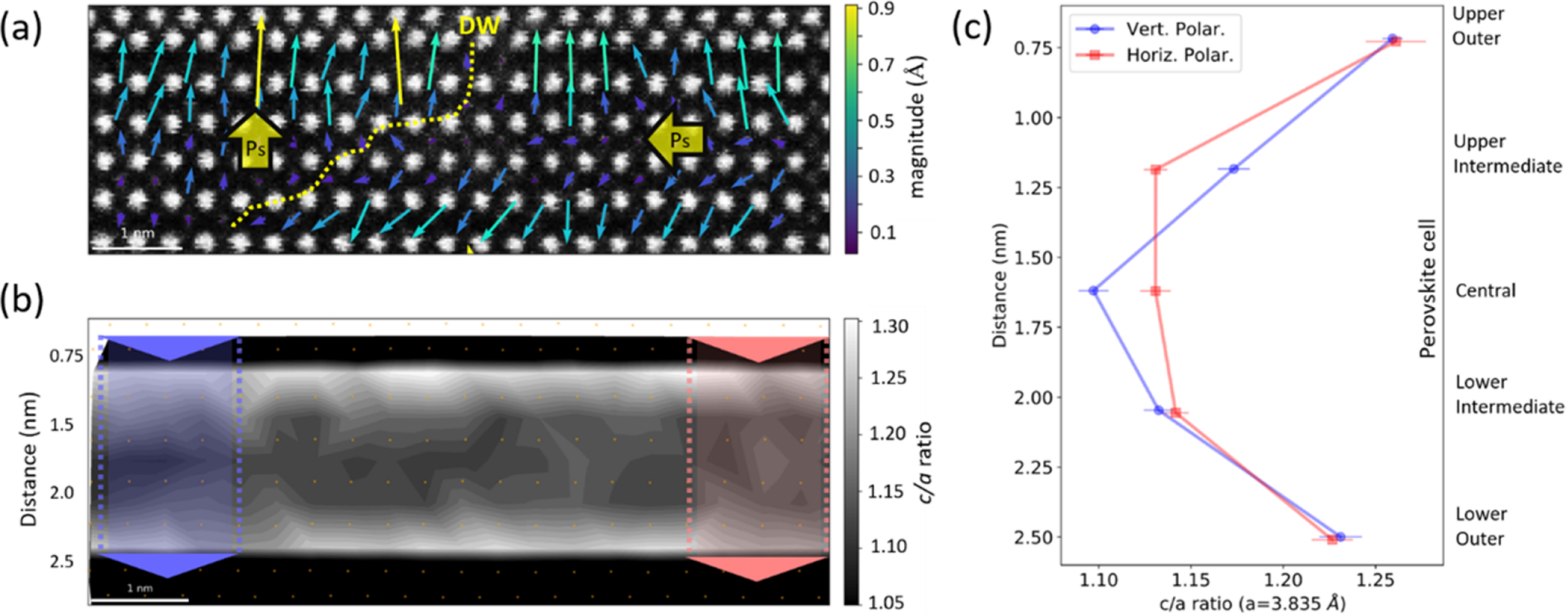
(a) Polarization map of a head-to-tail DW. Vectors are colored by the magnitude of the B-site displacement. Polarization magnitudes are suppressed in lower perovskite cells and enhanced in upper perovskite cells in the left domain. This results in a net vertical, *c*-axis polarization as indicated by the large yellow arrow in the left domain. (b) c/a ratio map of panels (a) and (c) graph comparing line profiles across the *c*-axis polarized “vertical” domain (blue) and the in-plane polarized “horizontal” domain (red) in panel (b). The shaded area between dotted lines marks the position of the profiles. Strain values in panel (c) are averaged over five perovskite cells, indicated by the arrow widths in panel (b). In panel (c), there is a clear asymmetry in the vertically polarized domain, demonstrating the link between strain and polarization in the structure. Error bars represent standard error of the mean. Scalebars = 1 nm.

In Figure 4a, a second type of DW is analyzed, where the net polarization is head-to-tail (H–T). There is a stark difference in configuration compared to the H–H DWs. First, the net polarization transition across the DW is sharp. Second, polarization magnitudes are suppressed in lower perovskite cells and enhanced in upper perovskite cells in the left domain. This results in a net vertical, *c*-axis polarization. H–T DWs are the most commonly observed type of DW in proper ferroelectrics. Usually, in odd-layered Aurivillius phase materials, perfect mirroring along the *c*-axis is not possible due to symmetry and octahedral rotations. The sole contributor to out-of-plane polarization is therefore expected to be the symmetry breaking central perovskite cell, with mirrored polarization of the outer and intermediate perovskite cells.^49−51^ What we observe in the left domain of Figure 4a is completely different; polarization is almost uniformly pointing up in every perovskite cell. The lower perovskite cells have a suppressed polarization magnitude (B-site displacement) ∼0.1 Å, while the upper perovskite cells have an enhanced polarization magnitude of ∼0.7 Å. For reference, the polarization magnitude in bulk BiFeO_3_ is ∼0.2 Å. Faraz et al. measured an out-of-plane ferroelectric response of 10 pm/V in these films.^16^

There is a clear correlation between this asymmetric *c*-axis polarization and the ε*_yy_* strain of the ferroelectric layer in Figure 4c. For the vertically polarized domain in Figure 4a, larger polarization vectors for the upper-outer and upper-intermediate perovskite cells correspond to increased ε*_yy_* strain in Figure 4c, compared with the in-plane polarized domain. While there is less ε*_yy_* strain in the central perovskite cell compared with the in-plane domain, there is no ε*_yy_* strain difference between vertical and in-plane domains for the intermediate and outer perovskite cells at the bottom of the layer, where the polarization is suppressed. For the horizontally polarized domain in Figure 4, both ε*_yy_* strain and polarization vectors are symmetrical. The difference in strain states between the two domain types means there is a relatively high anisotropy cost for the DW. This manifests itself in the sharp, well-defined, H–T transition across 1–2 perovskite cells, contrasting with the wider H–H DW in Figure 2 but still following the same angled orientation. From EELS mapping (Supporting Information Figure S5), there is a clear indication of oxygen depletion at the DW. Oxygen vacancies are common in perovskites and normally found even at H–T DWs purely due to the strain field present, without needing to provide electrostatic screening.^52,53^

**Figure 5:**
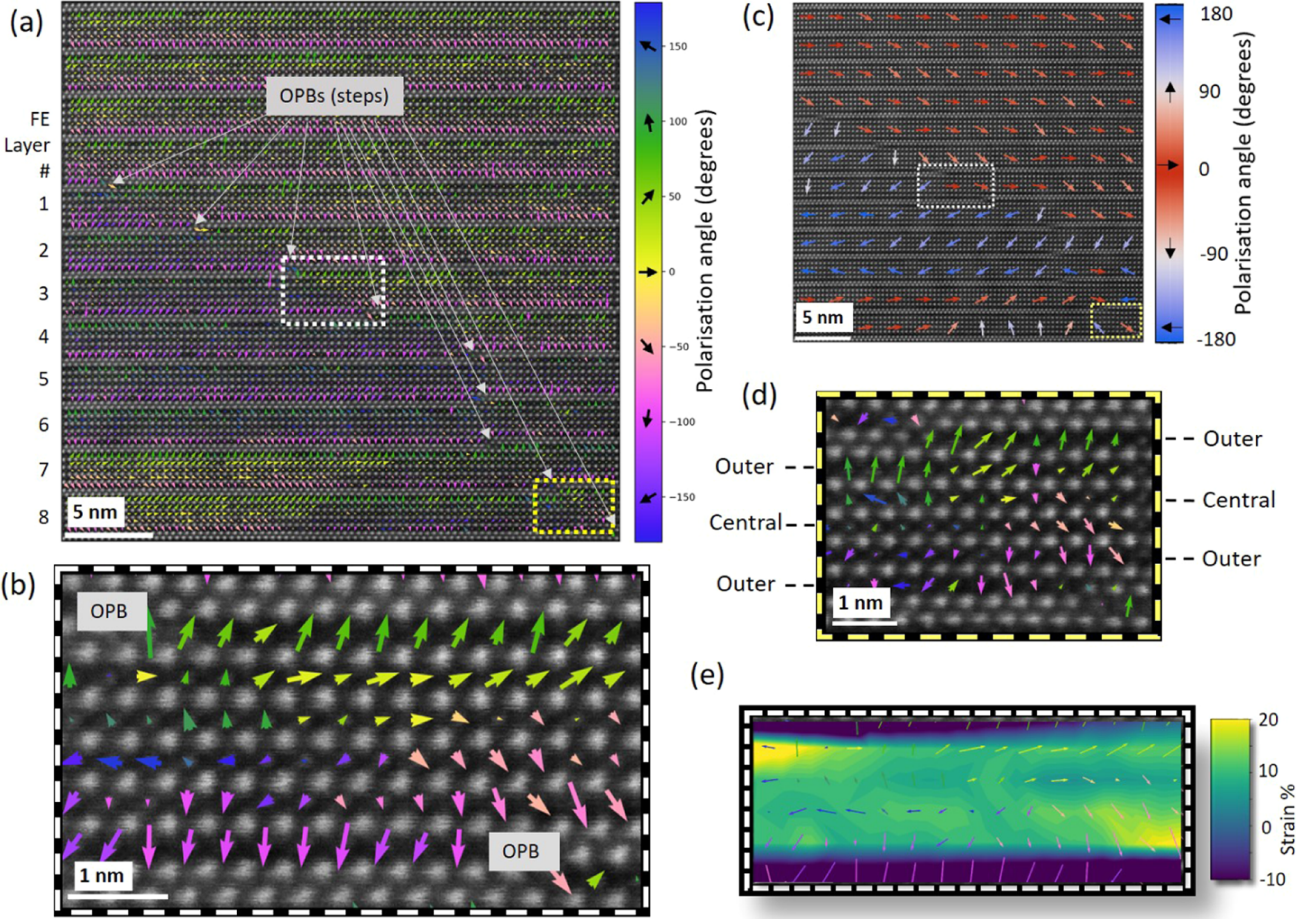
(a) Polarization map of a large area containing DWs pinned at OPBs. The polarization vector colors represent their angle relative to the *x*-axis. Two vortices are present, marked by dashed boxes. (c) Same map as panel (a), with polarization vectors summed over 5 × 5 perovskite cells and color-coded red/blue to demonstrate the T–T character of DWs. (b) Enlarged area from the white dashed box in panel (a) detailing the polarization vortex, which forms where T–T DWs occur in the vicinity of OPBs. (d) Enlarged area from the yellow dashed box in panel (a) where another vortex has formed. Perovskite cells are labeled to demonstrate the change in the structure at the OPB. (e) Polarization vectors from panel (b) overlaid on a ε*_yy_* strain map of the same area. ε*_yy_* strain is defined as the change of the *c* parameter from the average *a* parameter (3.835 Å), with 0% representing a pseudo-cubic structure. Panels (b) and (d) share the same polarization color bar as panel (a). See Supporting Information Figures S6–S8 for more details.

A further illustration of the interplay between strain and polarization is shown in Figure 5, where ferroelectric polar vortices form. Rotating polarizations of the vortices are mapped in Figure 5c,d. The vortex occurs as a form of 180° polarization transition between T–T domains. As such, the vortex must be charged to the same extent as the H–H DW in Figure 2, with average σ_s_ = 1.09 e per perovskite cell. Although the DW width is similar to the H–H case in Figure 2, the defined vortex polarization suggests the charge density is concentrated at the vortex core,^54^ further increasing the relative conductivity difference versus the domain.

Subunit cell defects, such as out-of-phase boundary (OPB) defects are a common occurrence in materials of high structural anisotropy, such as the Aurivillius phases and are characterized by displacement of a fraction of a lattice parameter (c/x) between two neighboring regions parallel to the *c*-direction.^55^ They appear as a “step” in the dielectric and ferroelectric layer and are energetically preferred to dislocations for relieving out-of-plane stress in the film. Disruptions of the Aurivillius phase lattice by OPBs have a marked influence on the internal elastic strain and electrostatic energy gradients within the structure and further drive larger cations away from the OPB defect toward the central layers of the structure.^17^ From Figure 5a, it is evident that the line of OPB defects propagates diagonally down through the film, similar to the preferred orientation of the H–H DW in Figure 2. Figure 5c, in which the polarization vectors are summed over 5 × 5 perovskite cells, shows that the T–T DW broadly follows the OPBs down through the layers of the film. The T–T DW decouples from the OPB defects in ferroelectric layers 5–7, which instead contain a 180° neutral DW and a 180° H–H DW. Ferroelectric layer 8 contains both a H–H DW and a T–T vortex. This is shown in detail in Figure 5d. The rich variety of features within just 20 nm is testament to the complex electrostatic screening in the film. As shown in the low-magnification images of Supporting Information Figure S9, OPB defects are found throughout different samples grown during different growth runs.

Vortices are more traditionally associated with ferromagnetic systems, where there is no anisotropy cost to extend the transition of the magnetization direction, thus lowering the exchange energy at the DW. Ferroelectric vortices have previously been identified in thin films of BiFeO_3_ and PbTiO_3_ multilayers^56,57^ but remain a novel occurrence. However, the coupling of multiferroic-order parameters is known to induce a chirality at DWs;^58,59^ thus, as this material is multiferroic and coupled to strain at the OPBs, it is, in fact, not surprising that vortices can form. To examine the strain state of the vortex, we compare the *c*-axis strain map (ε*_yy_*) in Figure 5e to the perovskite cell polarization in Figure 5b. There is an asymmetric ε*_yy_* strain gradient on either side of the vortex, but no strain change at its center. Thus, the anisotropy cost, DW width, and the exchange energy (determined by the curl of the polarization) should show the same behavior as the H–H DW in Figure 2. We reason that the vortex occurs due to geometric/strain effects controlling the polarization, as shown in Figure 5e. Increased ε*_yy_* strain to the upper-left and lower-right of the vortex core corresponds to OPBs adding an extra perovskite cell polarized up and down, on either side, i.e., the increased ε*_yy_* strain is caused by the “steps”. Furthermore, the central perovskite cells, which provide the in-plane polarization are displaced.

In Figure 5b,d, when the lower central perovskite cell on the left becomes the intermediate perovskite cell after the OPB, the polarization turns downward. When the upper central perovskite cell on the right becomes the intermediate perovskite cell after the OPB, the polarization turns upward. Thus, the 180° T–T DW, instead of behaving similar to the H–H DW in Figure 2, becomes a vortex with a well-defined polar rotation. In line with this explanation, we observe that the lateral distance between successive OPBs must be similar to the 180° charged DW width, 5–8 perovskite cells, for the vortex to form. The factors affecting vortex formation in each of the ferroelectric layers #1–8 in Figure 5a are detailed in Table 1.

**Table 1:** Comparison of the Factors Affecting Vortex Formation in the Ferroelectric Layers Containing OPBs Presented in Figure 5a,c

ferroelectric layer	charged DW present between OPBs?	OPBs spaced 5–8 perovskite cells apart?	vortex present?
1	yes	no	no
2	yes	no	no
3	yes	yes	yes
4	yes	no	no
5	no	no	no
6	no	no	no
7	no	yes	no
8	yes	yes	yes

Moving from the perovskite cell to the ferroelectric layer scale, there is evidence of polarization coupling through multiple ferroelectric layers in each of the multilayer maps. The dielectric layers do not fully isolate the ferroelectric layers. In the film, the polarization can rotate freely toward the viewing axis, adding an extra degree of freedom to the system. PFM results in Figure 1d–g and from other Aurivillius phase materials show that nominally charged DWs are evident in as-grown films,^7^ so the ferroelectric results from STEM HAADF presented here appear to be representative. Of course, as always, one should consider whether ferroelectric behavior may be altered in a cross-sectional STEM lamella compared to the as-grown thin film. Thin lamellae strongly favor polarization within the plane of the lamella to reduce surface charging. The conduction mechanisms in similar Aurivillius phase films vary depending on the strength of the applied field, but both ionic conductivity and electron hopping conductivity have an important contribution according to Song et al.^60^

These results offer a detailed insight into the ferroelectric behavior of ion-substituted Aurivillius phase thin films. However, at the nanoscale, there remain significant questions to be answered. For instance, the structure of Aurivillius phase materials offers intriguing hints pointing toward localized charge separation. Each ferroelectric layer has a sharp transition from up to down polarization across the five perovskite cells, and thus, one could consider this transition as a horizontal DW in the ferroelectric layer. The central perovskite cell should then contain positive screening charge, with the dielectric layer containing negative screening charge.^20^

In our previous work,^17^ we experimentally identified that Mn has a preference for concentrating toward the central perovskite cell and thus plays a key role generating room-temperature ferromagnetism. Similar atomic-scale STEM energy dispersive X-ray (EDX) spectroscopy mapping for this study was performed in the same OPB defect region as in Figure 5b and is presented in Supporting Information Figure S10. We observe an augmented partitioning of cations with a decreased positive charge (Mn with overall nominal valence of 3+) away from the OPB defect regions and there is a promoted concentration of lower valence cations at the central perovskite layers. Conversely, there is a promoted partitioning of cations with increased positive charge (Ti with presumed overall nominal valence of 4+) toward the defect regions and the outer perovskite layers. This indicates that OPB defects provoke electrostatic energy changes and that there is an increased electrical conductivity at the ferroelectric vortex core, similar to electric-field gradient observations for PbTiO_3_/SrTiO_3_ multilayers^54^ and BiFeO_3_.^61^ Mn^3+^, the nominal oxidation state in B6TFMO to achieve charge balance, is known to disproportionate into Mn^2+^ and Mn^4+^.^62^ The transition to Mn^2+^ and Mn^4+^ provides a likely source of electrons and holes for charge screening. Although outside the scope of this paper, charge separation between the center and edges of each ferroelectric layer is thus a potentially interesting phenomenon to be further investigated by atomic-scale fine structure EELS.

## Conclusions

In summary, we have demonstrated the formation of both H–H and T–T DW-type topologies within multiferroic B6TFMO thin films by cross-sectional STEM and top surface PFM experimental observations. DWs are coupled between ferroelectric layers and are not altogether electrostatically isolated by the dielectric layers in between. The DW topologies are highly charged with average σ_s_ = 1.09 |e| per perovskite cell and ρ ∼ 0.17 |e| per perovskite cell. The exact nature of screening charge is yet to be determined but local charge separation is likely due to the intrinsically opposing polarization within the ferroelectric layer. H–T domains exhibit *c*-axis polarization and the accompanying strain asymmetry signals electromechanical coupling. It is important to reemphasize that the multiferroic Aurivillius phase B6TFMO contains a naturally occurring dielectric–ferroelectric layered structure.

Second, we show that this material system is a rich landscape to explore nontraditional ferroelectric DWs such as chiral topologies and thus potential spin-to-charge coupling at such topologies. We show that OPB boundary defects induced complete 2D polar vortices, if separated by a distance similar to the DW width. The atomic-scale characterization of the vortices gives insight into the scale and magnitude at which locally induced elastic strain and electrostatic energy changes can influence polarization chirality. We hope this study relating to anisotropy and exchange energy at different DWs can inform and spur increased interest into the magnetoelectric coupling at chiral topologies formed in Aurivillius phases and other multiferroic thin films. These results highlight the B6TFMO Aurivillius phase as an ideal platform for multiferroic topology engineering and thus low-power electric-field-controlled magnetic device applications.

## Methods

### Crystal Growth

B6TFMO films were synthesized on *c*-sapphire substrates by liquid injection chemical vapor deposition (LI-CVD) methods^16^ and postannealed at 850 °C (1123 K). The average stoichiometry, as determined by high-resolution scanning electron microscopy (HR-SEM) with EDX, was Bi_6_Ti_3.04_Fe_1.42_Mn_0.54_O_18_.

### Electron Microscopy

Cross sections of B6TFMO films were prepared using a Thermo Fisher Scientific Dual Beam Helios NanoLab 600i and G4 model focused ion beam (FIB) and were mounted on a Cu-based TEM grid. The sample was thinned via the FIB Ga beam first at 30 kV and 93 pA, then 5 kV and 43 pA, and then 2 kV and 16 pA. The samples were then further thinned and polished to sub 30 nm using a Fischione 1020 Ar ion-based plasma cleaner prior to STEM imaging and EDX/EELS analysis. Energy filtered images acquired at 300 kV on a Thermo Fisher Scientific Titan TEM with the Gatan Tridiem Energy Filtering system demonstrated that thicknesses of the regions used for imaging were <30 nm. Imaging and analysis was performed on a NION UltraSTEM 200 operating at 200 kV and a Thermo Fisher Scientific Titan Themis operating at 300 kV. A Gatan Enfinium and GMS 2.0 was used for EELS acquisition and analysis. EDX analysis was performed using a Bruker 100 mm^2^ windowless EDX detector. EDX acquisition and analysis was performed using Bruker Esprit 2.0. Energy filtered images acquired at 300 kV on an FEI Titan TEM with Gatan Tridiem Energy Filtering system demonstrated that thicknesses of the regions used for imaging were <35 nm. Images were taken along the [110] crystallographic zone axis. EDX signal intensities for Ti, Fe, and Mn were used as a proxy for the relative proportions of each atom on each of the five PK layers in the structure, normalized to 100% B-site occupancy.

Atom position finding and 2D Gaussian refinement were completed with the Atomap Python package.^63^ Image analysis and mapping, as well as polarization vector analysis, were completed using the TopoTEM module^64^ of the TEMUL Toolkit Python package. Strain analysis was carried out by geometric phase analysis using Stem Cell.^65^

### Piezoresponse Force Microscopy

Electromechanical responses of the films were measured by PFM using an Asylum Research MFP-3D AFM in contact mode equipped with a HVA220 Amplifier for PFM and a cantilever scan angle of 90°. The Dual AC (alternating current) Resonance Tracking Piezoresponse Force Microscopy (DART-PFM) mode was used to boost both the vertical and lateral piezo signals. In this mode, the PFM signal is measured at the tip–sample contact resonance frequency, with a higher signal-to-noise ratio compared with other frequencies. Topographical cross-talk is reduced using an amplitude feedback loop, which tracks the contact resonance frequency so that drive frequencies are adjusted accordingly as the probe scans over the changing sample topography. Application of an AC bias to a conductive tip during contact mode imaging (*V*_tip_ = V_AC _cos(ω*t*)) results in surface displacement (*d*) and deflection of cantilever due to the converse piezoelectric effect, with both normal and in-plane components. Olympus AC240TM Electrilevers, Ti/Pt-coated silicon probes (Al reflex coated, 15 nm tip radius, 70 kHz resonant frequency), were used for PFM and topography imaging. The angular torsion of the cantilever as it oscillates was monitored in lateral PFM measurements. The drive frequencies were operated near contact resonance for lateral (670–720 kHz) and vertical (250–280 kHz) modes, respectively, with a probing signal of 1.0 V_AC_.

### Density Functional Theory Calculations

Density functional theory (DFT) calculations were carried out on 140-atom unit cells for the cation ordering calculations and 280-atom unit cells for the DW energetics calculations of Bi_24_Ti_11_Fe_6_Mn_3_O_72_. Our calculations used the Vienna *ab initio* simulation package (VASP)^66^ with projector augmented wave (PAW) pseudo-potentials^67^ and Perdew–Burke–Ernzerhof exchange correlational functionals.^68^ We treat Bi (6s, 6p), Ti (3d, 4s), Fe (3d, 4s), Mn (3d, 4s), and O (2s, 2p) as valence. We used a planewave cutoff energy of 600 eV and a Monkhorst–Pack ***k***-point grid of 4 × 1 × 4 for the 140-atom unit cell and 2 × 1 × 4 for the 280-atom unit cells. To correct for the known underlocalization of *d*-orbitals of Mn and Fe, we applied a Hubbard-U correction of 3 and 4 eV, respectively, consistent with previous works on transition-metal oxides,^69^ with the rotationally invariant version of GGA+*U* by Dudarev et al.^70^ We performed spin-polarized calculations to optimize the lattice parameters and internal coordinates until the forces on each atom were less than 0.01 eV Å^–1^. For all of our cation ordering configurations, we calculated the ferromagnetic ordering and three different antiferromagnetic orders, with the final lowest-energy magnetic order relaxing to a ferrimagnetic case for each, as depicted in Figure 3.

## Supporting Information Available

Representative data demonstrating multiferroic behavior of *m* = 5 Aurivillius phase samples; averagedpolarization plotting of STEM image of charged head-to-head domain wall; extended strain analysis of the head-to-head domain wall; polarization plotting and strain analysis of STEM image of head-to-tail domain wall; polarization plotting of STEM image of region with OPBs and vortex regions, strain profiles across the vortex in the εyy strain map, STEM imaging of a different thin film bulk samples showing OPBs throughout, polarization plotting with corresponding EDS elemental spatial mapping (PDF)
