# Supplementary material for: Charged Domain Wall and Polar Vortex Topologies in a Room-Temperature Magnetoelectric Multiferroic Thin Film
Source: ACS Appl Mater Interfaces. 2022 Jan 19;14(4):5525–36. doi: 10.1021/acsami.1c17383 (PMC8815039; doi:10.1021/acsami.1c17383)
Supplement: Supplementary file 1 — am1c17383_si_001.pdf [file am1c17383_si_001.pdf]

Supporting Information

Charged Domain Wall and Polar Vortex Topologies in a Room Temperature Magnetoelectric Multiferroic Thin Film

Kalani Moore,[1] Eoghan N. O'Connell,[1] Sinéad M. Griffin,[2,3] Clive Downing,[4] Louise Colfer,[5] Michael Schmidt,[5] Valeria Nicolosi,[4,6] Ursel Bangert,[1] Lynette Keeney,[5] Michele Conroy[1,7,8*]*

1. Department of Physics, Bernal Institute, School of Natural Sciences, University of Limerick, Limerick, V94 T9PX, Ireland

2. Materials Sciences Division, Lawrence Berkeley National Laboratory, Berkeley CA, 94720, USA

3. Molecular Foundry, Lawrence Berkeley National Laboratory, Berkeley, CA, 94720, USA

4. Advanced Microscopy Laboratory & AMBER, Trinity College Dublin, Dublin, D02 PN40, Ireland

5. Tyndall National Institute, University College Cork, Cork, T12 R5CP, Ireland

6. School of Chemistry, Trinity College Dublin, Dublin, D02 PN40, Ireland

7. Department of Materials, Imperial College London, Exhibition Road, London SW7 2AZ, U.K.

8. London Centre for Nanotechnology, Imperial College London, Exhibition Road, London SW7 2AZ, U.K.

*Corresponding author emails: m.conroy@imperial.ac.uk and lynette.keeney@tyndall.ie

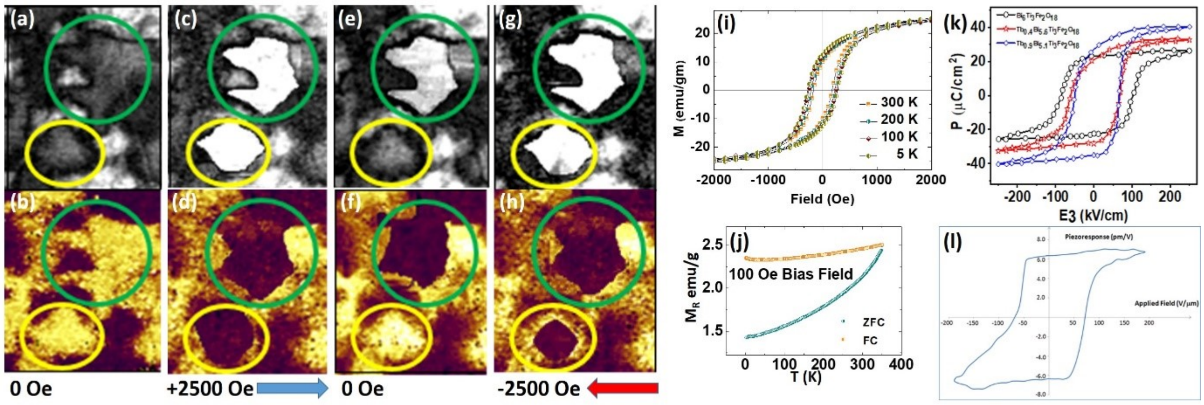

Figure S1: Representative data demonstrating multiferroic behaviour of m = 5 Aurivillius phase samples. **(a) - (h)** Irreversible (encircled green) and reversible (encircled yellow) magnetoelectric switching of ferroelectric domains in B6TFMO under a varied magnetic field. **(i)** Magnetization (M) vs magnetic field (Oe). **(j)** Zero field cooled (ZFC) and field cooled (FC) measurements (M_R vs T). The saturated hysteresis loops and clear split between ZFC-FC curves demonstrate the ferromagnetic nature of B6TFMO at RT. Adapted with permission from Faraz, A.; Maity, T.; Schmidt, M.; Deepak, N.; Roy, S.; Pemble, M. E.; Whatmore, R. W.; Keeney, L., Direct Visualization of Magnetic-Field-Induced Magnetoelectric Switching in Multiferroic Aurivillius Phase Thin Films. J. Am. Ceram. Soc. 2017, 100 (3), 975. Copyright 2016 John Wiley and Sons.[1] **(k)** Macroscopic in-plane polarisation (P) vs electric field (E_3) hysteresis loops obtained using interdigitated electrodes. Reprinted in part with permission from Faraz, A.; Ricote, J.; Jimenez, R.; Maity, T.; Schmidt, M.; Deepak, N.; Roy, S.; Pemble, M. E.; Keeney, L., Exploring Ferroelectric and Magnetic Properties of Tb-Substituted m= 5 Layered Aurivillius Phase Thin Films. Journal of Applied Physics 2018, 123 (12), 124101. Copyright 2018 AIP Publishing.[2] **(l)** Local out-of-plane switching spectroscopy piezoresponse force microscopy ferroelectric hysteresis loop. Reprinted in part with permission from Keeney, L.; Maity, T.; Schmidt, M.; Amann, A.; Deepak, N.; Petkov, N.; Roy, S.; Pemble, M. E.; Whatmore, R. W., Magnetic Field-Induced Ferroelectric Switching in Multiferroic Aurivillius Phase Thin Films at Room Temperature. Journal of the American Ceramic Society 2013, 96 (8), 2339-2357. Copyright 2013 John Wiley and Sons.[3]

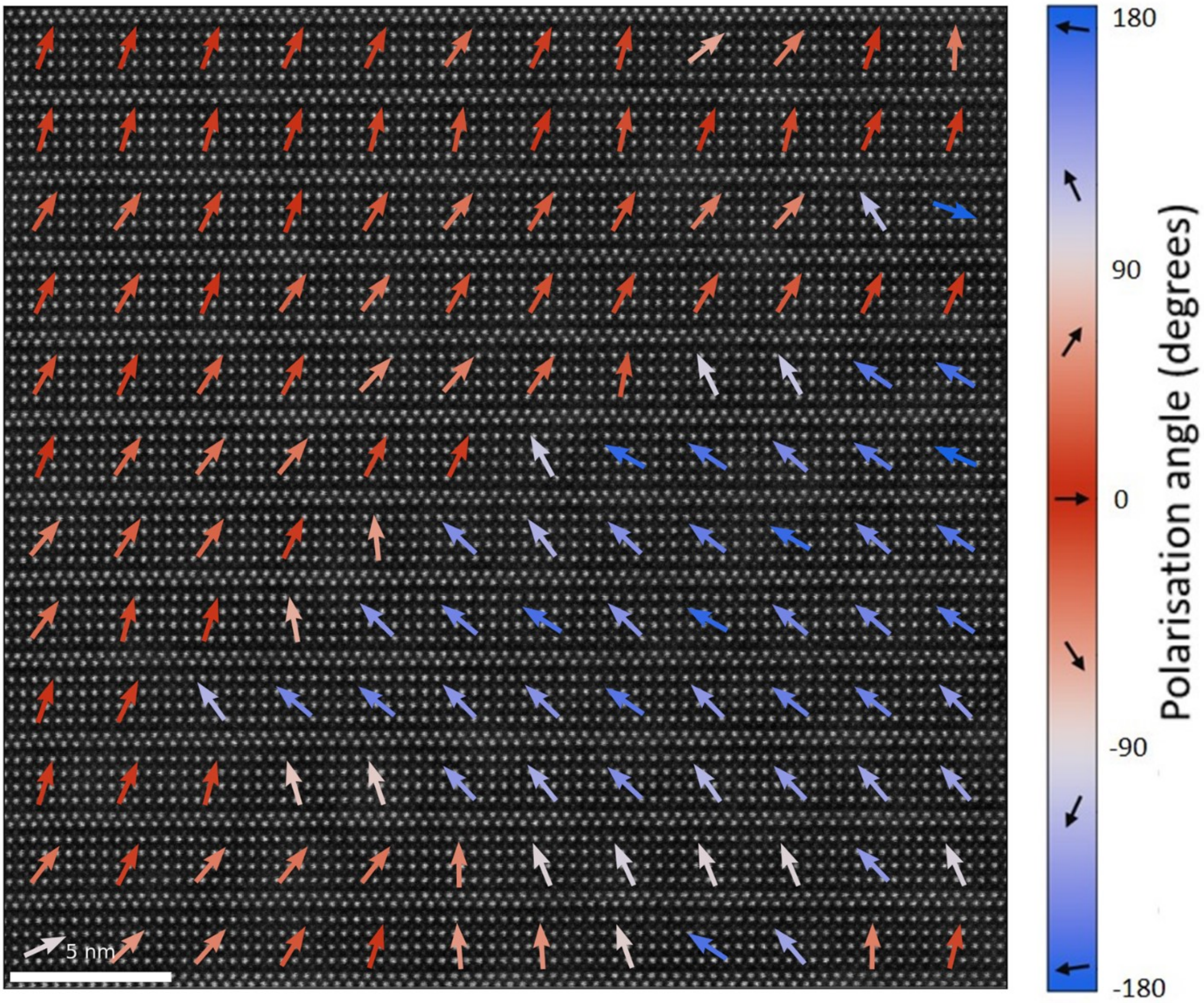

Figure S2: The same polarisation map as Figure 1(c) in the main article, but averaged over 5 x 5 unit cells. There is a significant upwards c-axis component to the measured net polarisation, which may be due to slight off-zone axis tilt in the sample. This map compares with Figure 1 (c), which shows averaged net polarisation almost completely in-plane, although there are some smaller areas of c-axis polarisation. Indeed the ability of B6TFMO to be polarised out-of-plane is demonstrated by the head-to-tail domain wall in Figure 2. Regardless of whether sample tilt, a true upward polarisation, or some combination thereof, is causing the out-of-plane appearance, the analysis of the head-to-head nature due to the in-plane polarisation components in Figures 1 & 2 remains applicable. Scalebar = 5 nm.

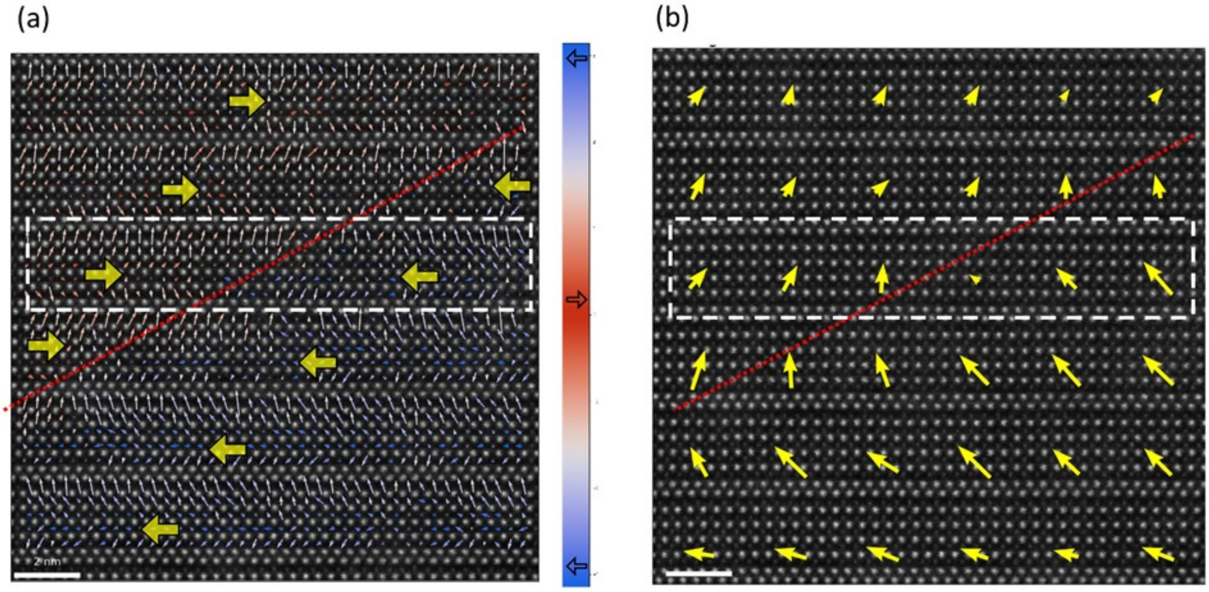

Figure S3: (a) Polarisation map of 6 perovskite (PK) layers with interpreted net polarisation indicated by yellow arrows. The layer contained by the dashed box is analysed in Figure 2 of the main paper. A 180° head-to-head (H-H) domain wall (DW) is present, connecting through the perovskite layers, indicated by the dotted red line. (b) The same polarisation map but with the polarisation vectors averaged over 5x5 PK-cells. There is a net upward c-axis component in addition to the in-plane opposing polarisation . The out-of-plane component may appear due to sample tilt. Scalebars = 2 nm.

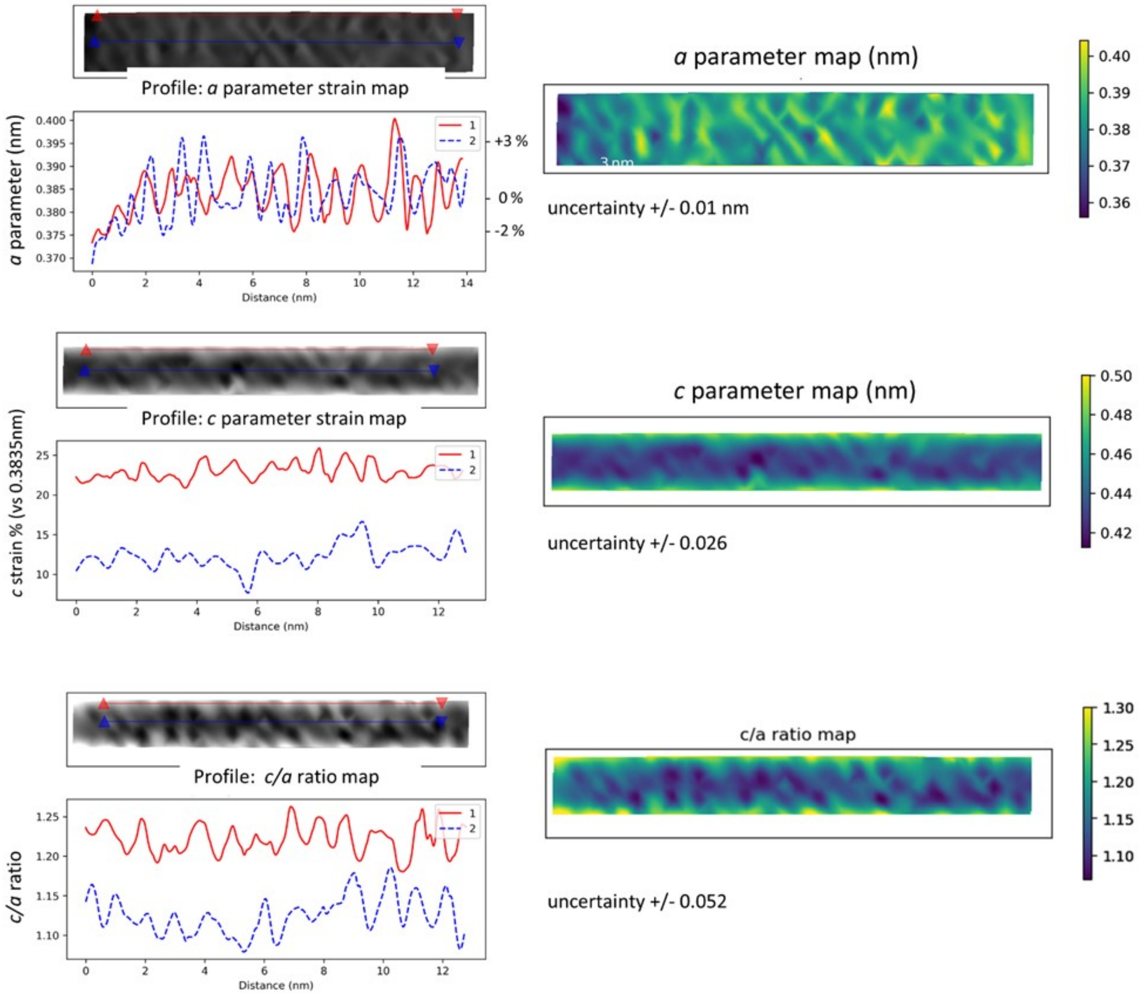

Figure S4: Extended strain analysis of the head-to-head domain wall in Figure 2 of the main paper. The uncertainty for the double-gaussian fitting method used varies with image signal to noise ratio. We have taken a value of 1/3 of a pixel for each atom. Distance/strain measurements use two atom locations so the uncertainty is 2/3*(0.15 Å per pixel) = 0.1 Å. The c/a ratio map combines two measurements, introducing more error and thus more variance in the map and profile. The same trend is identified as in the map and profile measured relative to the average a spacing in Figures 2(c & d).

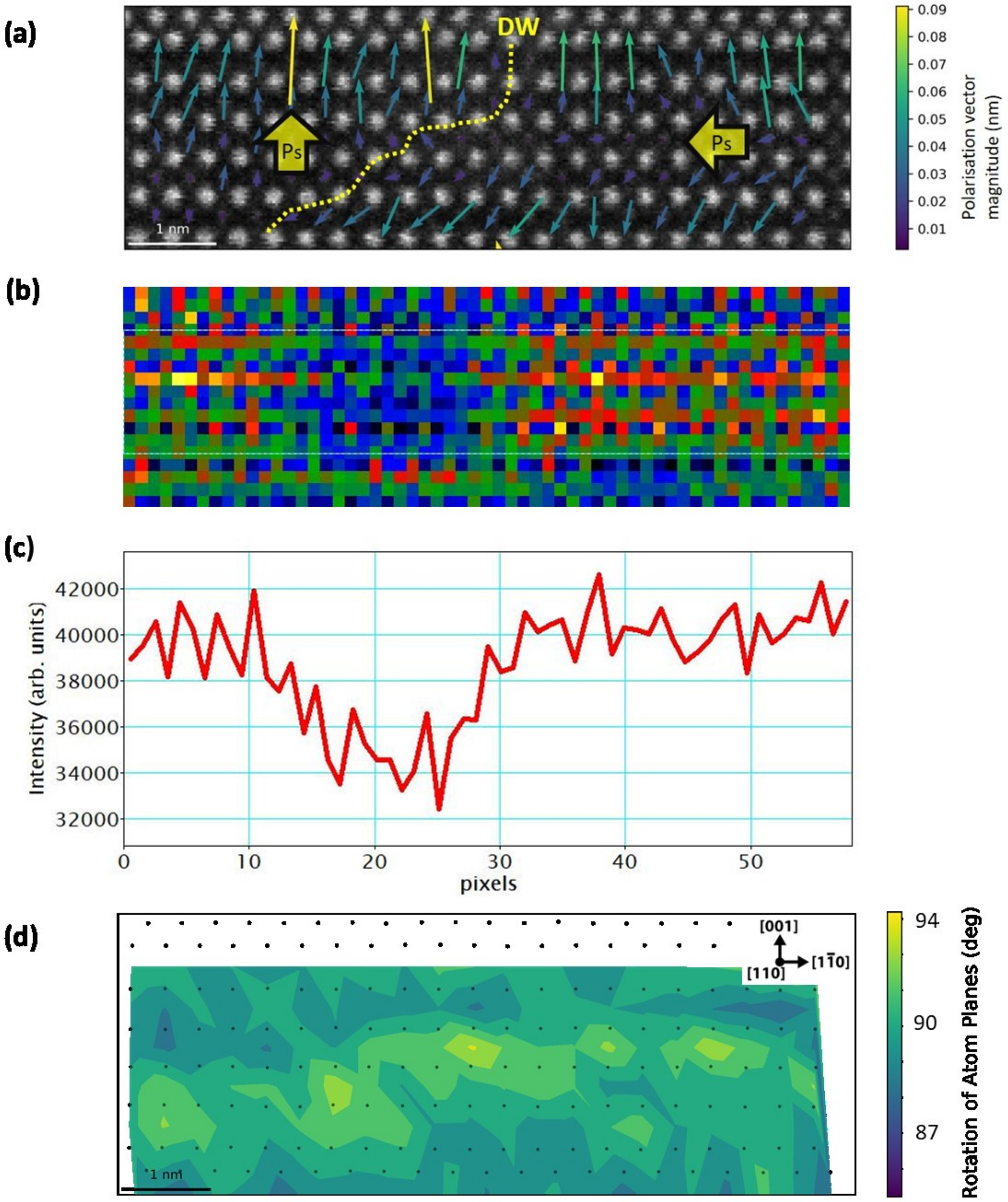

Figure S5: (a) Head-to-tail (H-T) domain wall (DW) shown in Figure 4. (b) Oxygen EELS core loss map of the same area as (a) extracted using a power law background and a signal window from 530 - 570 ev in Digital Micrograph. Principle components analysis was applied to the spectrum image before extraction. (c) Intensity profile across the map in (b). Integration width was 10 pixels to exclude the dielectric layer. The drop in the intensity profile and lower intensity in the map correspond to oxygen vacancies at the DW. (d) Rotation map of the [001] zone of the perovskite cells at the H-T DW. There is some indication of increased shearing at the DW. In comparison to the clear correlation seen at the H-H DW in Figure 2(b) the shearing does not seem to be confined to the H-T DW. The magnitude of rotation ~6˚ is again notably large.

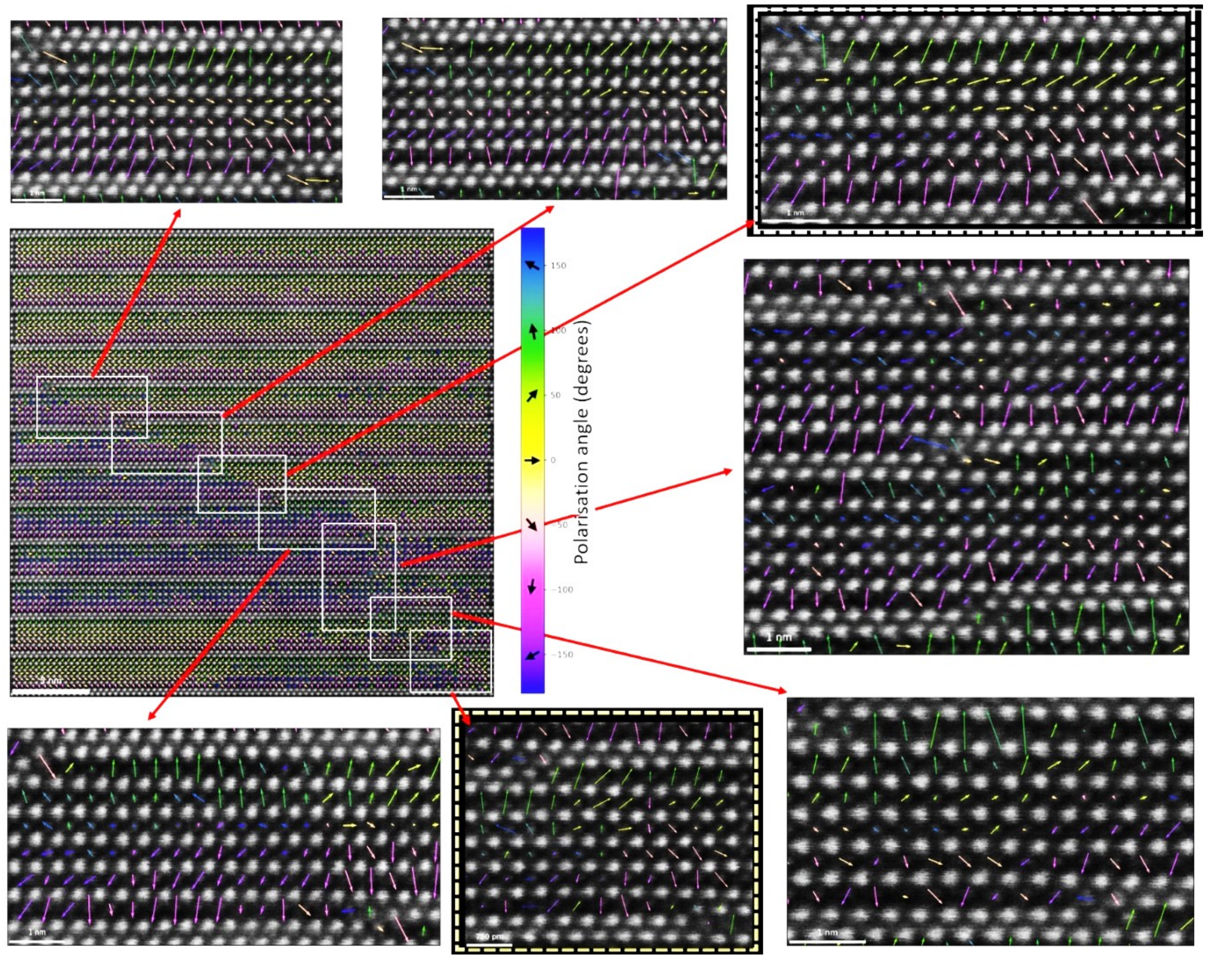

Figure S6: The same polarisation map as displayed in Figure 5. Scalebar = 5nm. Enlarged areas are portions of the map between successive OPBs, displayed for each PK-layer. The effects of the various DWs and OPBs listed in table 1 can be seen in the differing behaviour of the polarisation in each PK-layer. White and yellow dashed boxes surround the areas included in Figure 5(c) and Figure 5(d) respectively in the main paper.

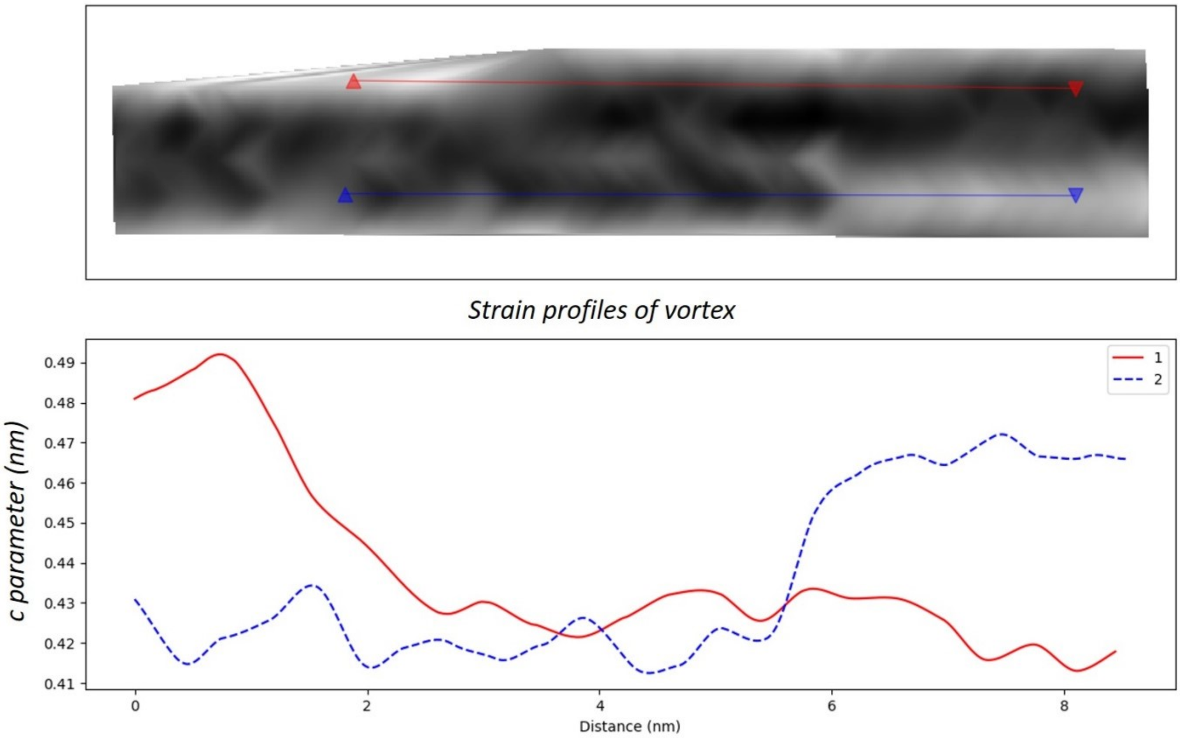

Figure S7: Strain profiles across the vortex in the ε_{yy} strain map displayed in Figure 5(e).

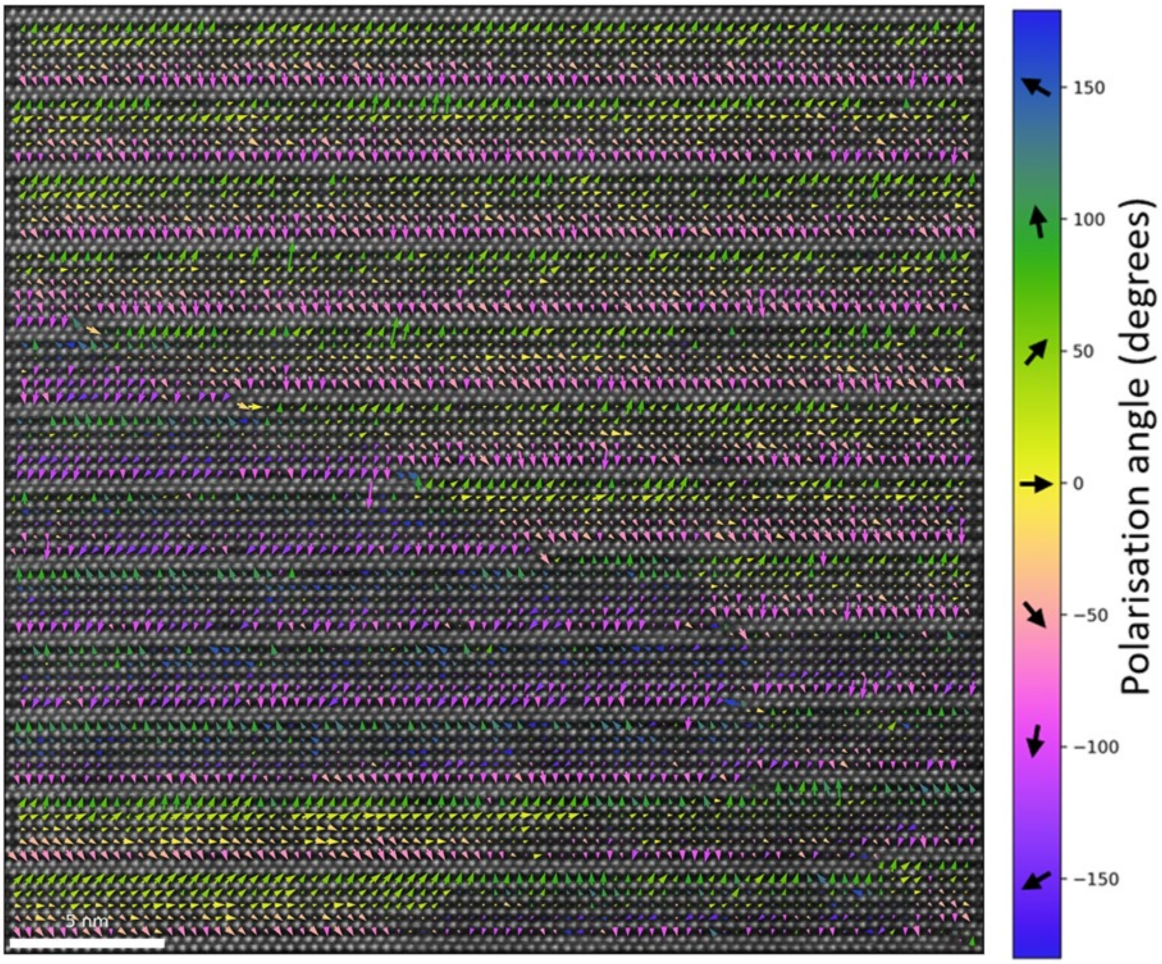

Figure S8: Enlarged view of Figure 5(a) to enable detailed inspection of each layer Scalebar = 5nm.

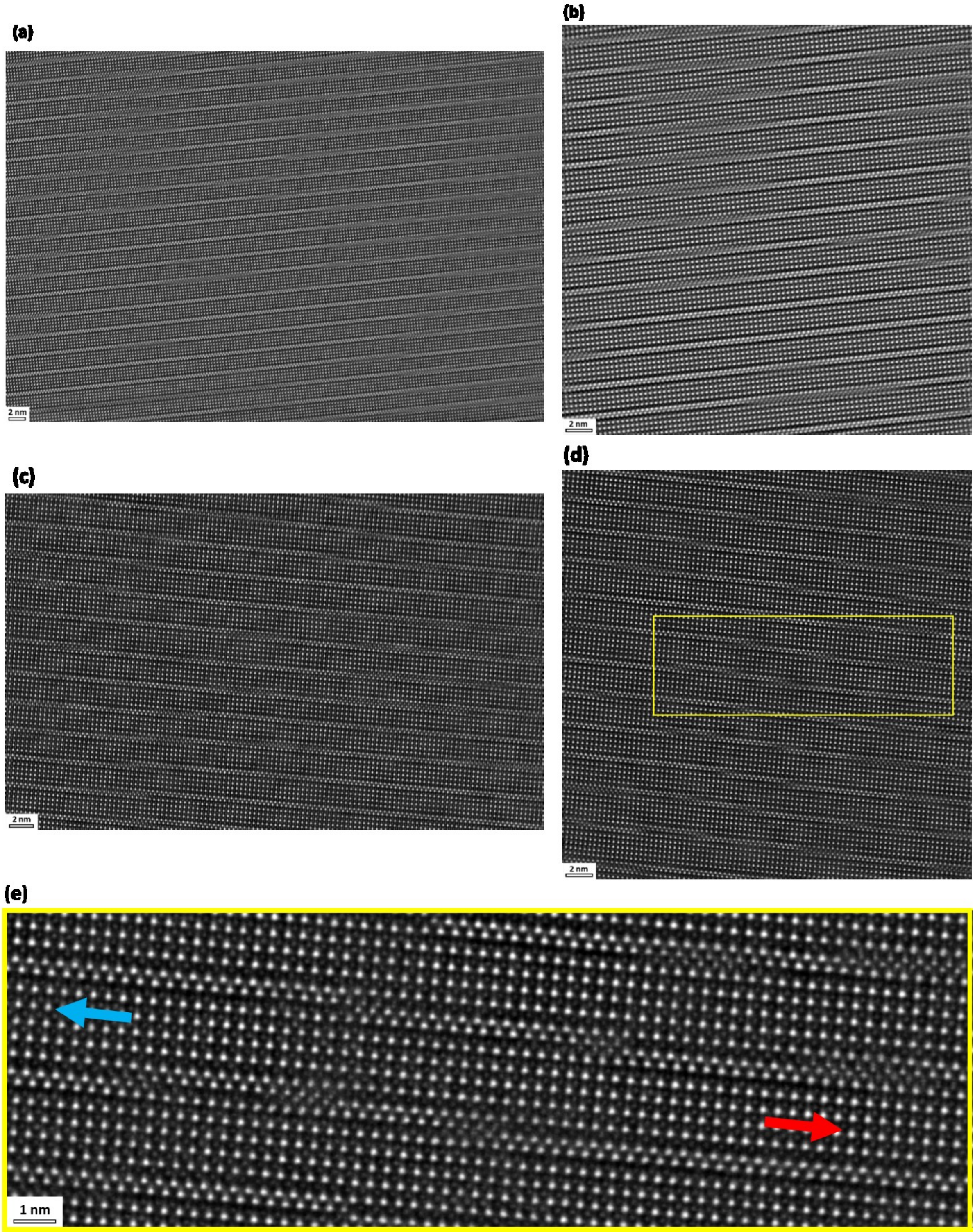

Figure S9: STEM HAADF imaging of a different thin film bulk sample. (a) and (b) FIB sample 1, (c) and (d) FIB sample 2,(e) higher magnification of OPB region highlighted in yellow of (d).

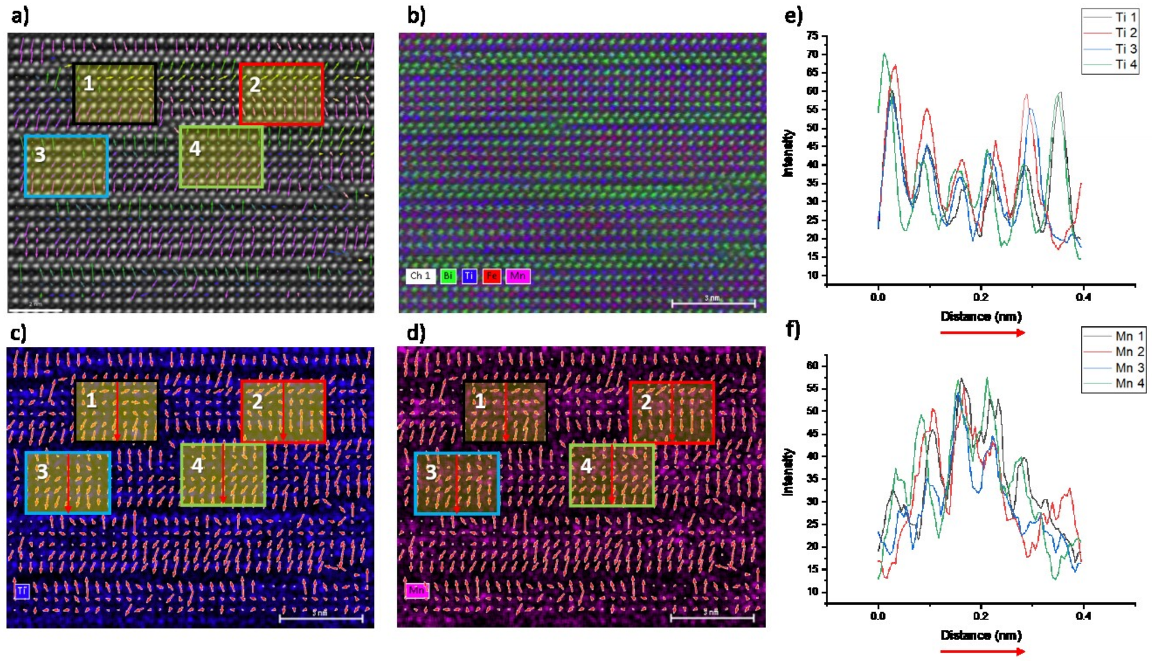

Figure S10: (a) Polarisation map of STEM HAADF image. (b) Atomic resolution EDX chemical composition map of the same region as (a) where Bi = green, Ti = blue, Mn = pink, Fe = red. (c) Ti map with overlay of polarisation map. d) Mn map with overlay of polarisation map. Region 1 corresponds to the region highlighted by the white box in Figure 5 (c) of the main article. This region contains 6 perovskite layers (m = 6) and contains a domain wall and polar vortex pinned between two out-of-phase boundary (OPB) defects located at top left and bottom right of the area. Regions 2 and 3 are free from OPB and stacking fault defects and possess 5 perovskite layers (m=5). Region 4 has an m = 6 structure but only one OPB defect present at the top left of the structure. No DW and no polar vortexes are present in Regions 2, 3 and 4. Brightness intensity profiles in the direction of the red arrows in (c) and (d) are plotted in terms of (e) Ti signal and (f) Mn signal, respectively for the 4 different regions. The Ti signal at the central layers is decreased further in Region 1, whereas there is an increased Mn signal at the central layers for Region 1.

SUPPORTING INFORMATION REFERENCES

1. Faraz, A.; Maity, T.; Schmidt, M.; Deepak, N.; Roy, S.; Pemble, M. E.; Whatmore, R. W.; Keeney, L., Direct Visualization of Magnetic-Field-Induced Magnetoelectric Switching in Multiferroic Aurivillius Phase Thin Films. *J. Am. Ceram. Soc.* **2017,** *100* (3), 975.

2. Faraz, A.; Ricote, J.; Jimenez, R.; Maity, T.; Schmidt, M.; Deepak, N.; Roy, S.; Pemble, M. E.; Keeney, L., Exploring Ferroelectric and Magnetic Properties of Tb-Substituted m= 5 Layered Aurivillius Phase Thin Films. *Journal of Applied Physics* **2018,** *123* (12), 124101.

3. Keeney, L.; Maity, T.; Schmidt, M.; Amann, A.; Deepak, N.; Petkov, N.; Roy, S.; Pemble, M. E.; Whatmore, R. W., Magnetic Field-Induced Ferroelectric Switching in Multiferroic Aurivillius Phase Thin Films at Room Temperature. *Journal of the American Ceramic Society* **2013,** *96* (8), 2339-2357.
